# Supplementary material for: Scrutinizing XAI using linear ground-truth data with suppressor variables
Source: Mach Learn. 2022 Apr 13;111(5):1903–23. doi: 10.1007/s10994-022-06167-y (PMC9123083; doi:10.1007/s10994-022-06167-y)
Supplement: Supplementary file 1 — Supplementary Information. [file 10994_2022_6167_MOESM1_ESM.pdf]

Scrutinizing XAI using linear ground-truth data with suppressor variables (Supplementary Material)

Rick Wilming · Céline Budding ·
Klaus-Robert Müller · Stefan Haufe

November 2021

Rick Wilming
Technische Universität Berlin, Germany
E-mail: rick.wilming@tu-berlin.de

Céline Budding
Eindhoven University of Technology
E-mail: c.e.budding@tue.nl

Klaus-Robert Müller
Technische Universität Berlin, Germany
BIFOLD – Berlin Institute for the Foundations of Learning and Data, Berlin, Germany
Korea University, Seoul, South Korea
Max Planck Institute for Informatics, Saarbrücken, Germany
E-mail: klaus-robert.mueller@tu-berlin.de

Stefan Haufe
Technische Universität Berlin, Germany
Physikalisch-Technische Bundesanstalt Berlin, Germany
Charité – Universitätsmedizin Berlin, Germany
E-mail: haufe@tu-berlin.de

S1. Experiment 2: stronger overlap between signal and distractor

The results presented in the main text are obtained using signal and distractor components that are overlapping in the top left corner of the image, whereas the signal is also present in the bottom left corner, and the distractor is also present in the top right corner. Here we study a slightly more challenging setting where the signal is *only* present in the top left corner, while the distractor pattern remains the same (see Figure S1). A classifier thus cannot leverage the (largely) unperturbed signal from the bottom left corner, but needs to extract the class-specific information from the top left corner. In order to remove interference from the distractor, a stronger influence of the top right corner (suppressor) is necessary.

Figure S2 shows that the studied classifiers can deal with this case as well, although the achieved classification accuracies are slightly lower than for the case studied in the main text.

Figures S3–S6 demonstrate that the results obtained for this setting are comparable to those obtained for the setting studied in the main text. For those XAI methods negatively affected by suppressor variables, the performance according to the PREC90 metric, however, drops significantly. This can be attributed to the stronger overlap of the signal and distractor components and the resulting increased difficulty of the explanation task. The decrease in terms of AUROC is less pronounced, which we attribute to the higher class imbalance in Experiment 2 compared to the setting studied in the main paper. As AUROC is positively biased by the degree of imbalance, we also study the average precision as a less biased metric (see supplementary Section S2.), which also show a larger drop in explanation performance compared to the setting studied in the main paper.

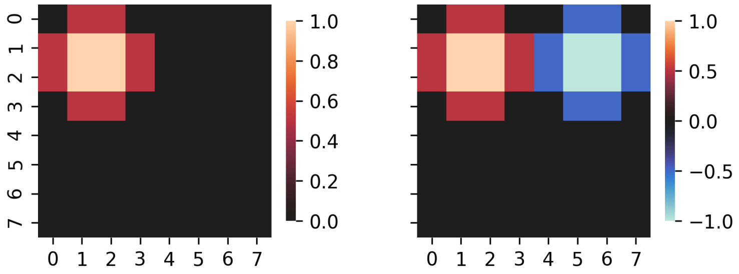

Fig. S1 The signal activation pattern **a** (left) and the distractor activation pattern **d** (right) used in our experiments. In contrast to the main text, the signal is now only present in the upper left corner and is, thus, completely superimposed by the distractor.

S2. Average Precision

Since, in the setting of Experiment 2, the important features are underrepresented compared to the unimportant features (8:56), we consider the average precision (AVGPREC) as an alternative to the AUROC performance metric, which is known to be biased towards higher values in this imbalanced situation. The AVGPREC

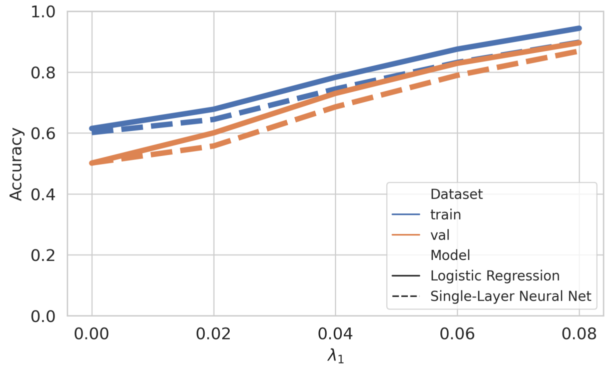

Fig. S2 With increasing signal-to-noise ratio (determined through the parameter λ_1 of our generative model, the classification accuracy of the logistic regression and the single-layer neural network increases. Since, the signal is more strongly superimposed with the distractor compared to the setting studied in the main text, the observed small drop in accuracy can be expected.

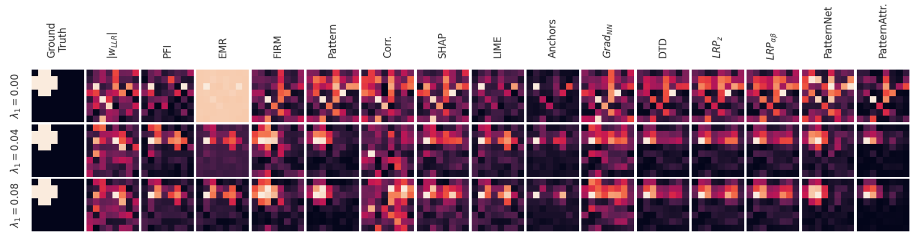

Fig. S3 Global saliency maps obtained from various XAI methods on a single dataset. Here, the 'ground truth' set of important features, defined as the set of pixels for which a statistical relationship to the class label is modeled, is the upper left blob. Also in this setting, a number of XAI methods assign significant importance to pixels in the right upper part of the image, which are statistically unrelated to the class label (suppressor variables) by construction.

summarizes the precision-recall curve as the mean of the precision values per threshold, weighted by the increase in recall from the preceding lower threshold

$$\text{AVGPREC} := \sum_i P_i (R_i - R_{i-1}), \tag{1}$$

with P_i and R_i are the precision and recall values at the i-th threshold.

S3. Correlation between LLR and NN weight vectors

Figure S8 shows the correlation between the model weight vectors estimated by LLR and the neural-network based implementation for five different signal-to-noise ratios. As the neural network based implementation has two output neurons, the effective weight vector, $\mathbf{w}^{\text{NN}}$, was calculated as the difference $\mathbf{w}^{\text{NN}} = \mathbf{w}_1^{\text{NN}} - \mathbf{w}_2^{\text{NN}}$. Although not identical, both weight vectors are found to be highly correlated.

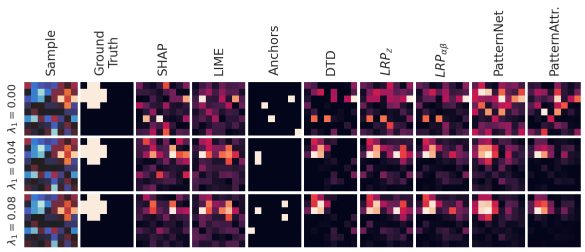

Fig. S4 Saliency maps obtained for a randomly chosen single instance. At high SNR, DTD, PatternNet and PatternAttribution best reconstruct the ground truth signal pattern, while SHAP, LIME, Anchors, and LRP assign importance to the right half of the image, where no statistical relation to the class label is present by construction

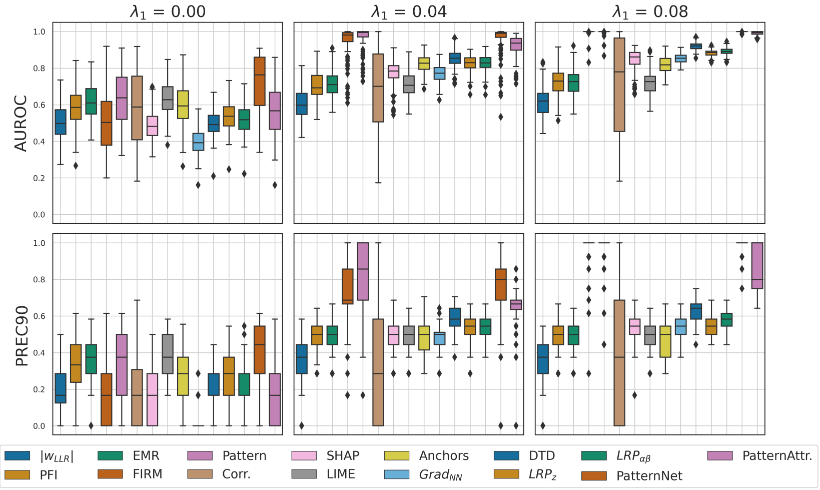

Fig. S5 Within the global XAI methods, the linear Pattern and FIRM consistently provide the best 'explanations' according to both performance metrics. For $\lambda_1 = 0.08$, DTD also reaches a high AUROC score. Note, however, that AUROC is a less suitable metric in this setting compared to the setting studied in the main text, because the number of truly important features is much smaller than the number of unimportant features. Therefore, the PREC90 metric as well as the average precision (see supplementary Section S2.) are more suitable metrics. Among the instance-based methods, the saliency maps obtained by PatternNet and PatternAttribution (averaged across all instances of the validation set) show the strongest explanation performance, where PatternAttribution exhibits high variance for $\lambda_1 = 0.08$ in the PREC90 metric.

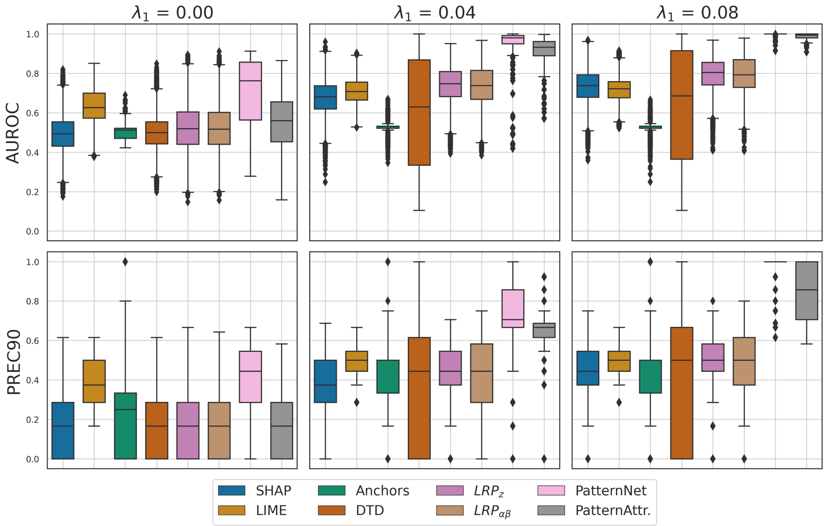

Fig. S6 Explanation performance of instance-based saliency maps of neural-network-based XAI methods. For medium and high SNR, PatternNet and PatternAttribution show the strongest capability to assign importance to those feature that are associated with the classification target by construction, while ignoring suppressor features.

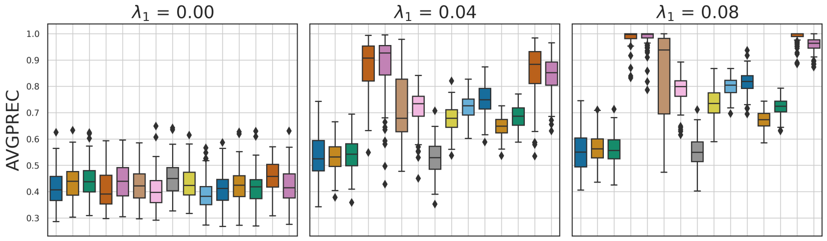

(a)

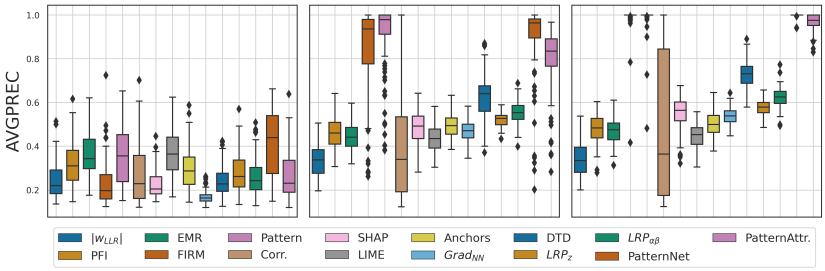

(b)

Fig. S7 Average Precision of global explanations for the two-blob signal pattern used in the main manuscript (a) and of the single-blob signal pattern used in Experiment 2 (see Figure S1) (b). Since we have a stronger overlap between signal and distractor, all scores decrease in general. But also according to this metric, the linear Pattern, Firm, PatternNet and PatternAttribution attain the highest scores.

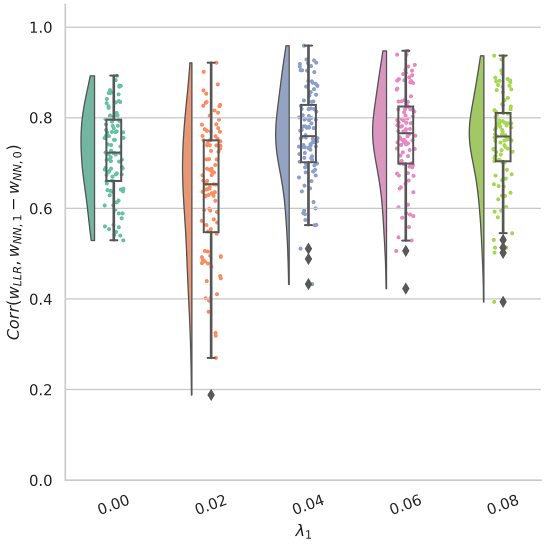

(a)

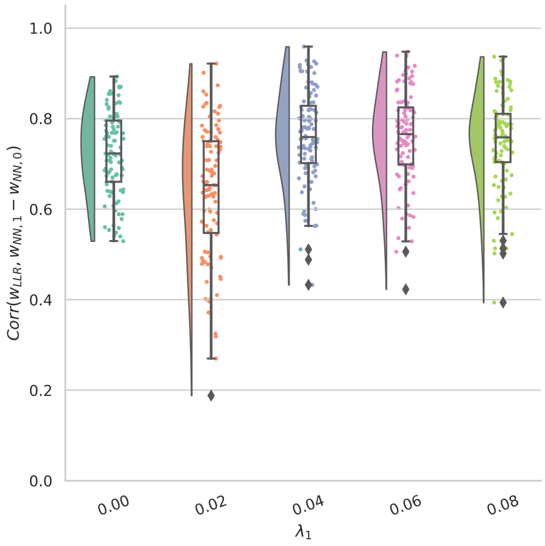

(b)

Fig. S8 Pearson correlation of the model weights of LLR and NN.
